# Self-Reported Stomach Upset in Travellers on Cruise-Based and Land-Based Package Holidays

**DOI:** 10.1371/journal.pone.0083425

**Published:** 2014-01-10

**Authors:** Naomi J. Launders, Gordon L. Nichols, Rodney Cartwright, Joanne Lawrence, Jane Jones, Christos Hadjichristodoulou

**Affiliations:** 1 Gastrointestinal, Emerging and Zoonotic Infections Department, Public Health England, London, United Kingdom; 2 EU Shipsan Trainet Project, Public Health Program, European Agency for Health and Consumers, Luxembourg, Luxembourg; 3 Federation of Tour Operators, London, United Kingdom; 4 Travel and Migrant Health Section, Public Health England, London, United Kingdom; 5 Department of Hygiene and epidemiology, Medical Faculty, University of Thessaly, Larissa, Greece; 6 Norwich Medical School, University of East Anglia, Norwich, United Kingdom; The Australian National University, Australia

## Abstract

**Background:**

International travellers are at a risk of infectious diseases not seen in their home country. Stomach upsets are common in travellers, including on cruise ships. This study compares the incidence of stomach upsets on land- and cruise-based holidays.

**Methods:**

A major British tour operator has administered a Customer Satisfaction Questionnaire (CSQ) to UK resident travellers aged 16 or more on return flights from their holiday abroad over many years. Data extracted from the CSQ was used to measure self-reported stomach upset in returning travellers.

**Results:**

From summer 2000 through winter 2008, 6,863,092 questionnaires were completed; 6.6% were from cruise passengers. A higher percentage of land-based holiday-makers (7.2%) reported stomach upset in comparison to 4.8% of cruise passengers (RR = 1.5, p<0.0005). Reported stomach upset on cruises declined over the study period (7.1% in 2000 to 3.1% in 2008, p<0.0005). Over 25% of travellers on land-based holidays to Egypt and the Dominican Republic reported stomach upset. In comparison, the highest proportion of stomach upset in cruise ship travellers were reported following cruises departing from Egypt (14.8%) and Turkey (8.8%).

**Conclusions:**

In this large study of self-reported illness both demographic and holiday choice factors were shown to play a part in determining the likelihood of developing stomach upset while abroad. There is a lower cumulative incidence and declining rates of stomach upset in cruise passengers which suggest that the cruise industry has adopted operations (e.g. hygiene standards) that have reduced illness over recent years.

## Introduction

Travel by land, air or sea puts the traveller at risk of infectious microorganisms not usually encountered in their home country and brings with it the potential to transport infectious diseases from one area to another [1], [2]. Gastrointestinal illness is common in travellers, with one study of package holiday tourists reporting travellers' diarrhoea in 24% of travellers questioned [3]. This may be even higher in those travelling from high to low income countries [4]–[6]. On cruises, respiratory illness has been shown to be the most common reason for visits to the ship's infirmary, followed by gastrointestinal illness which account for 9–11% of all visits [6], [7]. British Tour operators have developed a questionnaire-based means of monitoring stomach upsets in people returning from different countries [8].

Travellers' diarrhoea is generally defined as three or more loose stools in 24 hours, with at least one accompanying symptom (i.e. nausea, vomiting, abdominal cramps, tenesmus, bloody stools or fever), starting during or soon after international travel. Although many episodes of travellers' diarrhoea are self-limiting, prolonged diarrhoea is estimated to occur in 3% of travellers to regions of high risk [9]. As well as causing illness and distress for the traveller, and reducing the enjoyment of foreign holidays, travellers' diarrhoea also impacts on the country of residence. For example it is estimated that 50% of Shigella cases and 20% of Salmonella cases in England, Wales and Northern Ireland are travel-related [10], however, travel history is under-reported. Travel data has also been used to examine disease burden in countries visited [11]. The term stomach upset is undefined and passengers record their response to this depending on their understanding of the term.

Bacterial diseases, such as enterotoxigenic or enteroaggregative *E*. coli [12] and campylobacter [13], are the most common cause of travellers' diarrhoea. However, on cruise ships, norovirus is the most common cause of outbreaks. Although the United States Centres for Disease Control and Prevention Vessel Sanitation Programme (VSP) estimated that the chance of suffering gastrointestinal illness on a seven-day cruise is less than 1% [14], cruise ship outbreaks have been the subject of much media attention, leading to a perception that cruise ship holidays present a high risk of gastroenteritis.

The published literature on stomach upset on passenger ships is limited. There are studies based on investigations by ships doctors [7], [15], and the CDC VSP has a gastrointestinal illness surveillance system for cruise ships travelling in US waters. These studies are based on the ships doctor being aware of cases, possibly underestimating the problem. The relative risk for cruise ship passengers compared to those on land-based package holidays has not been investigated.

In this study we examine information from a Questionnaire that is designed for assessing customer satisfaction, but which also asks about illnesses acquired while abroad.

## Methods

A major British tour operator regularly administers a Customer Satisfaction Questionnaire (CSQ) to travellers over the age of 16 and resident in the UK, on the return flight of their holiday. The flights targeted for this process are defined by market research needs, and therefore are not random. Passengers were invited to complete a market survey questionnaire that includes a few simple questions about their health on the holiday they are returning from. Data on cruise passengers are predominantly from those who flew to a destination outside the UK to start their cruise. However, from 2005, data were also collected from passengers who had taken a cruise departing from the UK. Data from all questionnaires were entered into a database by a market research company.

The CSQ health data was introduced in the early 1980s as a tool to identify holiday destinations with above average levels of gastrointestinal disturbances among tourists, and tour operators present the results to the authorities responsible for high incidence destinations and encouraged authorities to take remedial actions such as the instigation of food hygiene programmes and the construction of new drinking water and waste water plants. The on-going nature of the CSQs enables the success or otherwise of remedial actions to be assessed, and is an integral part of the Federation of Tour Operators programme to promote improvements in health and safety within destination resorts.

The questionnaire contains questions regarding traveller demographics (age, sex, employment status, occupation), details about their holiday (hotel, resort, region, hotel chain, date of arrival/return, season, length of stay, type of board), customer satisfaction questions, and questions on self-reported illness (stomach upset for >24 hours, sea sickness, colds, sunburn or other illnesses). Definitions for these terms are not provided on the CSQ questionnaire, and are assumed to be common terms understood by the general public. It is assumed that some data will represent questionnaires from the same person travelling on more than one holiday and the analyses are therefore reported as passenger visits rather than populations, and not therefore a cohort.

Cruises were of variable lengths whereas the majority of land based package holidays were predominantly one or two weeks in duration, although some land based holidays were also of variable lengths. The codes used to denote length of stay changed between land, cruise, ski and other types of holiday but there was insufficient information to verify if the coding was correct. It was therefore not feasible to reliably compare land and cruise based holidays by length of stay.

Data from a total of 10,401,470 questionnaires completed during the 12 year period from May 1997 through April 2009 were received from the market research company. Questionnaires completed prior to May 2000 were excluded due to methodological differences, but the relevant questions were unchanged from then. Due to an operational problem (loss of a CSQ file), no data were available for May 2003. The extracted CSQ data was examined as records of foreign visits representing an unspecified percentage of all foreign visits involving a flight. It is understood that some travellers may have completed more than one CSQ if they had travelled on more than one occasion. While use of the surveillance data has been reported previously its limitations are not fully understood [8].

To determine those records related to cruise travel, the hotel names were searched for instances of “cruise” or “CRU”. The hotel names were also manually searched and compared with a list of cruise names used by the tour operator. Records were defined as “land-based only”, “cruise travel” or “unknown”. A total of 1,314 records were rejected as the type of travel could not be determined.

Data were analyzed using SPSS, STATA and Microsoft Excel. Denominator data for the estimated number of holiday visits abroad made by British residents were sourced from the Office for National Statistics International Passenger Survey (IPS) [16]. Data were available per quarter from the IPS. Where data were required for partial quarters an estimate was made assuming the rate of travel was the same throughout that quarter. The season of travel was defined using the date of return, with the summer season defined as May to October and winter season as November through to April of the following year.

Multivariable logistic regression was performed to examine rates of stomach upset with respect to cruise and land travel. All available variables were included in the initial model. Variables which were not significantly associated (p = 0.05) with stomach upset in univariate analysis and those with poor response rates (<80%) were removed sequentially, until the best fitting model was generated (backwards regression).

### Ethics statement

Data was from voluntarily completed questionnaires. None of the contributers can be identified or contacted.

## Results

### Customer Satisfaction Questionnaires (CSQs)

A total 6,863,092 CSQs completed from May 2000 (summer 2000) through April 2009 (winter 2008) were accepted for analysis, including 451,387 from cruise passengers (6.6%). Although the participation rate on flights is not known, these records are thought to represent 3.9% of the estimated 174 million package holiday visits abroad made by British residents during that time [16]. Numbers of completed CSQs varied seasonally, with peaks during the summer months for both land-based and cruise-based holiday makers corresponding to the seasonality of trips.

No information was available on the cruise itineraries; however the embarkation port for the majority of cruises was in Spain (62.2%), with 40.2% of all cruises beginning in Majorca, followed by Egypt (13.1%), Greece (13.1%) and the UK (5.8%). Land-based holidays had a larger range of destinations than cruise ship holidays, although the most commonly visited country was also Spain (55.6%), followed by Greece (13.4%), Cyprus (7.0%), Portugal (3.3%) and Egypt (2.9%).

The median age group of respondents who had been on cruise-based holidays (55–64 years) was higher than those on land-based holidays (45–54). A higher proportion of cruise passengers were men (48.7%) compared to those on land-based holidays (40.7%). More cruise passengers were retired (22.2% vs. 14.6%), or working as professionals (20.9% vs. 16.9%) than their land-based counterparts (Table 1).

**Table 1:** Demographic details of travellers on cruise- and land-based holidays.

	Cruise		Land		
	Count	95% CI	Count	95% CI	p-value†
**Number of holiday-makers:**	451387		6411705		
**Median age group:**	55–64		45–54		
**Gender:** % Men	48.68	48.53–48.83	40.66	40.62–40.7	<0.0005
**Social status:**					<0.0005
% Unskilled	6.65	6.57–6.73	7.84	7.81–7.86	
% Office/skilled	25.07	24.93–25.2	30.85	30.81–30.89	
% Technical/middle management	9.31	9.22–9.4	10.81	10.78–10.84	
% Professional	20.85	20.72–20.98	16.92	16.89–16.95	
% Student	11.59	11.48–11.69	12.32	12.29–12.34	
% Retired	22.15	22.01–22.28	14.57	14.54–14.59	
% Unemployed	0.84	0.81–0.87	1.21	1.2–1.22	
% House wife/husband	3.55	3.49–3.61	5.48	5.47–5.5	

Please note that because the sample size is very large and therefore even small effects will appear significant we have chosen P<0.0005 as a cut-off value compared to p values used in other smaller studies (i.e. P<0.01).

### Disease frequencies on land-based versus cruise-based holidays

Of the cruise passengers completing CSQs, 20.9% reported illness, compared to 16.2%, (p<0.05, Risk Ratio (RR) 1.3, CI 1.29–1.3) of those on land-based holidays. However, 10.2% of illness on cruise-based holiday was attributable to seasickness. When seasickness was removed from the analysis, the proportion of respondents reporting illness following cruise ship travel was lower than following land-based holidays (11.7% vs. 16.2%, (p<0.05, RR 0.8, CI 0.79–0.8)). Land-based holiday makers reported a higher proportion of stomach upset, sunburn, fractures and other illnesses than cruise passengers, whereas cruise passengers reported a higher proportion of colds (Table 2). While there were statistically significant differences for these, only stomach upset and sunburn had differences that were large enough to be important.

**Table 2:** Disease frequency in travellers on land- and cruise-based holidays.

Illness*	Land - % ill	Cruise - % ill	Risk Ratio	95% CI	p-value†
Fracture	0.01	0	N/A	N/A	N/A
Cold	2.34	2.45	0.96	0.94–0.98	<0.0005
Sunburn	3.96	1.39	2.85	2.78–2.93	<0.0005
Stomach upset	7.23	4.77	1.51	1.49–1.54	<0.0005
Sea sickness	0.00	10.2	N/A	N/A	N/A
Other	4.32	3.87	1.11	1.1–1.13	<0.0005

Travellers could report more than one illness. Sea sickness was not reported from land based holidays.

Please note that because the sample size is very large and therefore even small effects will appear significant we have chosen P<0.0005 as a cut-off value compared to p values used in other smaller studies (i.e. P<0.01). Our judgement is that the differences between land and cruise based are significant for cold, sunburn and stomach upset are significant, but the difference is probably not big enough to be important for colds, whereas it is for sunburn and stomach upset.

### Determinants of risk for stomach upset

**Figure 1:**
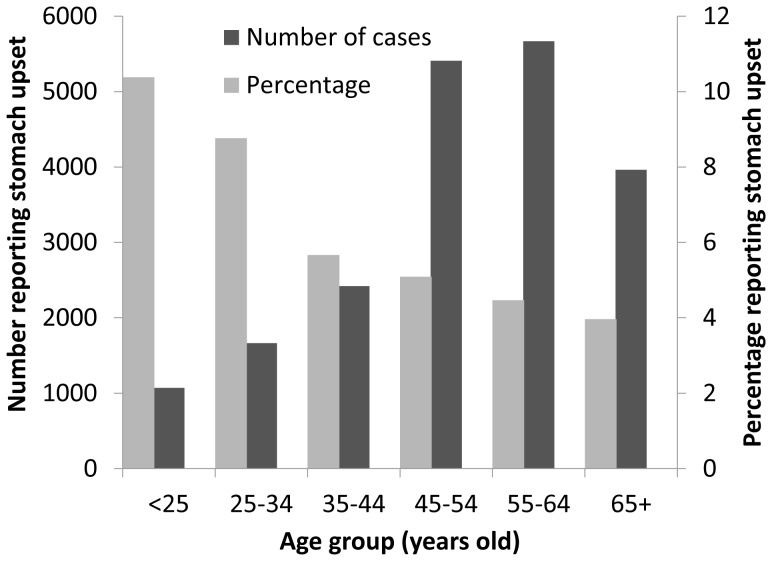
Age distribution of reported stomach upset on cruises.

The highest proportions of stomach upset were reported in those under the age of 25, with a steady decline in reporting in travellers over this age. However, for cruise ship passengers 74% of cases were reported in those 45 years of age or over (Figure 1).

**Figure 2:**
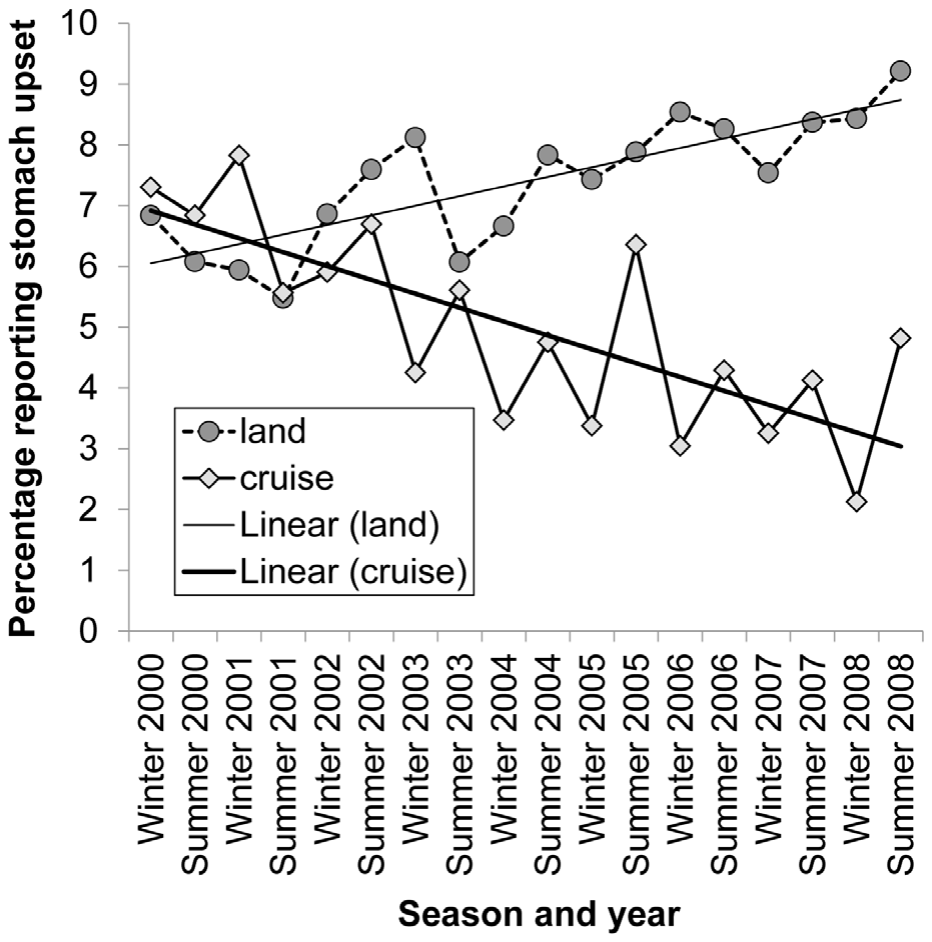
Trends in reports of stomach upset over time. Where S = Summer and W = Winter. Regression line for cruise-related stomach upset per year: R^2^ = 0.8984; regression line for land-based holiday-related stomach upset: R^2^ = 0. 7686.

A higher proportion of respondents who had been on land-based holidays reported stomach upset than those who had been on cruise-based holidays (7.2% vs. 4.8%; p<0.05, RR 1.5, CI: 1.49–1.54). The proportion of cruise passengers reporting stomach upset decreased over the study period (2000 7.1% (CI 6.9–7.3) vs 2008 3.1% (CI 3.0–3.2)2008 (p<0.05) (Figure 2). The frequency of stomach upset in travellers on land-based holidays increased from 2000 to 2008 (p<0.05). For cruise ship passengers, the reporting of stomach upset peaked during the winter months of each year, whereas for travellers on land-based holidays the seasonal pattern was less pronounced (Figure 2).

### Stratification by country

For all countries where CSQs were received for more than 100 cruise passengers, the risk of stomach upset for cruise-based travellers was significantly lower than that for land-based travellers to the same country, with the exception of the United States where no significant difference was seen (Table 3). The largest difference between the proportion of travellers reporting stomach upset on land- and cruise-based holidays was seen in travellers to the Dominican Republic (Land 27.9% vs. Cruise 3.3% (p<0.05, RR 8.6, CI 7.59–9.68).

**Table 3:** Incidence of stomach upset on land- and cruise-based holidays to countries for which >100 CSQs were received from cruise passengers.

	Land	Cruise	Risk Ratio	95% CI	p-value†
Country^a^	No. of cases (%)^c^	No. of cases (%)^c^			
Turkey	16048 (13.3)	33 (8.8)	1.50	1.08–2.08	0.0116
Spain	197641 (5.5)	9790 (3.5)	1.59	1.56–1.62	<0.0005
Greece	42880 (5.0)	1522 (2.6)	1.94	1.85–2.04	<0.0005
Cyprus	20385 (4.6)	140 (3.2)	1.41	1.2–1.67	<0.0005
Portugal	9240 (4.4)	126 (3.2)	1.35	1.14–1.61	0.0006
UK Cruise^b^	N/A	436 (1.7)			
Egypt	49352 (26.6)	8759 (14.8)	1.80	1.76–1.84	<0.0005
United States	2328 (4.2)	17 (3.7)	1.13	0.71–1.81	0.5986
Dominican Republic	38436 (27.9)	252 (3.3)	8.57	7.59–9.68	<0.0005
Barbados	609 (5.4)	181 (4.0)	1.37	1.16–1.61	<0.0005
Thailand	436 (12.7)	287 (6.6)	1.93	1.68–2.23	<0.0005

^a^ : Country of cruise departure for cruises, country of holiday stay for land-based holidays. It is expected that most cruises will stop at many ports and allow shore trips, so the departure country may not reflect the exposure risks on the cruise.

^b^ : UK cruise data only available from 2005 onwards.

^c^ : Cases of stomach upset (and as a % of total passenger trips.

Please note that because the sample size is very large and therefore even small effects will appear significant we have chosen P<0.0005 as a cut-off value compared to p values used in other smaller studies (i.e. P<0.01).

**Figure 3:**
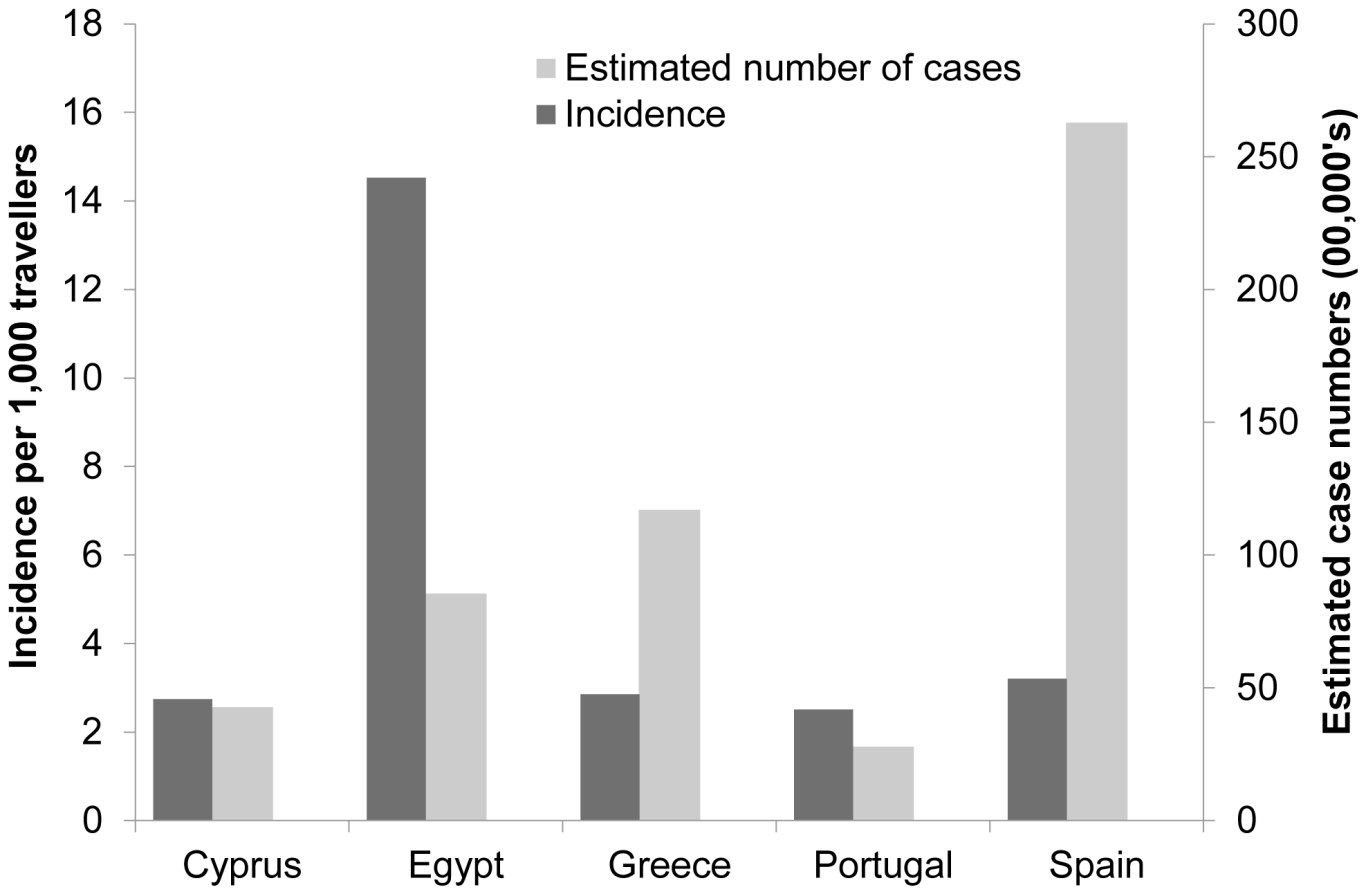
Incidence and estimated number of cases of stomach upset in travellers (both cruise- and land-based) in the period from Jan 2001 to Dec 2008.

While over 25% of travellers on land-based holidays to Egypt and the Dominican Republic reported stomach upset, in cruise ship passengers the highest proportion of stomach upset was 14.8% in travellers to Egypt, followed by 8.8% in travellers to Turkey (Table 3). Travellers to Greece and Spain reported a low cumulative incidence of stomach upset; however, due to the volume of travellers to these countries, the estimated number of cases in travellers was high. For countries such as Egypt, the opposite was observed, with a relatively high cumulative incidence but low number of cases (Figure 3).

**Figure 4:**
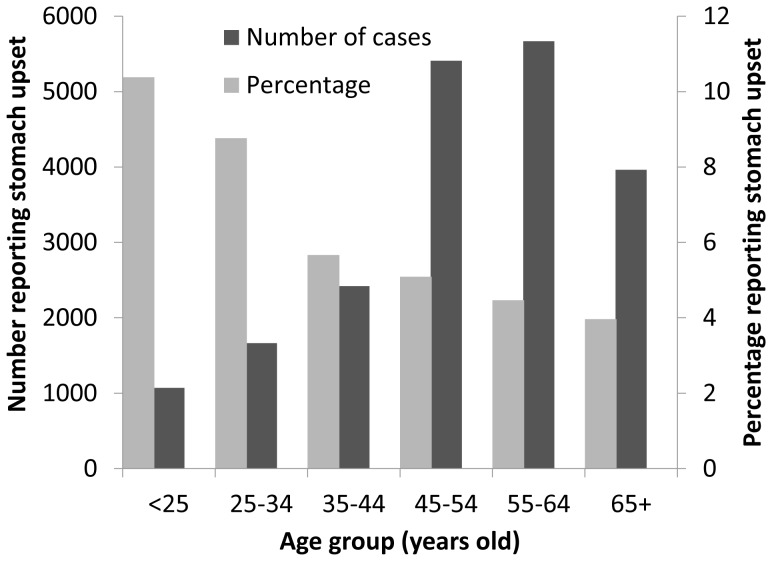
Seasonal distribution of prevalence of reported stomach upset in cruise passengers on cruises departing Spain and Egypt. S = Summer and W = Winter.

Over the study period, reporting of stomach upset decreased in cruise passengers to both Egypt (31.8% in 2000 vs 13.8% in 2008, p<0.05, r^2^ = 0.7903) and Spain (4.6% in 2000 vs 1.8% in 2008, p<0.05, r^2^ = 0.7896). The reporting of stomach upset in land-based travellers to Spain also decreased, but to a lesser extent (5.9% in 2000 to 4.8% in 2008, p<0.05, r^2^ = 0.5266), whereas no significant decrease was observed in travellers on land-based holidays to Egypt. With the exception of 2001, the incidence of stomach upset in cruises departing from Spain peaked in winter months every year, with 3.3% of all cruise passengers to Spain travelling in the summer seasons reporting stomach upset compared with 3.9% in those travelling during winter (p<0.05, RR 0.8, CI 0.8–0.9). For cruises departing from Egypt, 31.8% of passengers reported stomach upset during summer months compared to 9.8% during winter months (p<0.05, RR 3.2 CI 3.1–3.4) (Figure 4).

The risk of stomach upset varied greatly within each country. In Spain, for hotels and cruise ships where more than 1,000 CSQs had been completed, 1.7 to 25.3% of travellers to a particular hotel/cruise reported stomach upset (median = 5%), compared with 3.7 to 41.3% for travellers to Egypt (median = 25.1%).

### Multivariable analysis

In the multivariable logistic regression model, travellers on land-based holidays were more likely to report stomach upset, and those travelling in the summer months and earlier in the study period were also at increased risk. However, the model was a poor fit for the data (Homer-Lemshow test: p<0.0005), suggesting that the risk of stomach upset was not fully explained by these variables.

## Discussion

This is a large study of self-reported travel-related stomach upset, but as with any such study, it cannot be assumed that illness directly relates to rates of laboratory diagnosed infection, and in addition some misclassifications may have occurred. For travellers on cruise-based holidays, travellers may have misclassified stomach upset (including norovirus infection) and sea-sickness. However, sea sickness is generally short lived, and therefore the time aspect of the definition of stomach upset (>24 hours) should remove some of this misclassification. Because stomach upset is a colloquial term suggesting nausea and epigastric pain there is a question of whether people will report diarrhoea and vomiting as an upset stomach. The view we took was that amongst people in the UK returning from holiday the box would most likely be ticked for upset stomach in most passengers who had experienced diarrhoea and/or vomiting. There could also be misclassification through stomach upset recorded by passengers including dyspepsia in addition to diarrhoea and vomiting. There is no record of the percentage of passengers completing the survey, and because CSQ completion was voluntary there could be biases related to who completes such forms. The CSQ data will probably under-ascertain illness because many people will only develop symptoms after the return flight. However, despite these caveats and limitations previous evidence suggests that the analysis of this data is useful and informative [8] and the CSQ data provide records of over six million questionnaires completed by individual travellers over a nine-year period. An additional area that may cause inaccuracy is that cruise holidays may be shorter than land-based holidays. While it would have been nice to estimate the incidence of stomach upset per day of the holiday the data did not allow this to be done in a way that accurately reflected risk.

Our study found lower levels of stomach upset in package holiday travellers (7.2%) than previously reported (25.7%) [3] [4] [5]. Unlike our study these considered a broader range of traveller and we think that it is difficult to directly compare our results to these studies. This large retrospective study provides an interesting comparison of self-reported illness on cruise- and land-based holidays, and highlights the utility of alternative datasets in epidemiology. In this study, both demographic factors and holiday choice factors were shown to play a part in determining the likelihood of developing stomach upset while abroad. The finding that destination is the main determinant of illness reinforces the message that to improve traveller's health, the sanitation and health within destination countries must be improved. Further elucidation of the risk factors for acquiring a travel-related illness will allow travel health practitioners to tailor their advice for each traveller, and for travellers to make informed choices about their holiday destinations. The lower incidence and declining rates of stomach upset in cruise ship passengers over the nine years study period suggest that cruise industry measures to improve hygiene have reduced self-reported stomach upset, although the data does not indicate what these changes are.

Cruise ships passengers are seen as at high risk for gastrointestinal illness, in part due to the media interest in large norovirus outbreaks. Successful surveillance and reporting systems such as that run by the Centers for Disease Control Vessel Sanitation Program [17], [18] may also alter the perception of risk, providing a better surveillance mechanism than for many land-based locations. Although outbreaks on cruise ships with high attack rates have been reported [19], [20], this study suggests the proportion of travellers reporting stomach upset is lower in cruise ship passengers than for those on land-based package holidays when sea sickness is excluded.

As with other studies [8], [10], [21], [22], our study showed an increased risk of stomach upset when travelling outside of Europe and North America. The level of public health infrastructure, including access to clean drinking water and adequate sewage facilities, will affect the risk of stomach upset for travellers to that country [8], particularly for land-based travellers who are more exposed to a country's infrastructure. Cruise passengers staying on land as part of their holiday or participating in on-shore excursions in countries with poor public health infrastructure are also at risk, as are passengers of cruise ships which are using food and water purchased in such countries. As well as the risks posed by poor public health infrastructure, the risk of stomach upset is also dependent on the hygiene levels of individual resorts, hotels and cruises, as shown in the high variability in stomach upset rates between resorts or cruises in an individual country. This is particularly true in the package holiday market, where travellers may consume the majority of their meals in the hotel or on the cruise.

Our study has shown that the rates of self-reported stomach upset on cruise ships show strong seasonality. The different seasonal patterns in Egypt and Spain may be due to a predominance of different aetiological agents on the two types of cruises. The majority of reported outbreaks on large cruise ships in high income countries are due to norovirus [23], [24]. Incidence of norovirus peaks in winter months and may account for the winter peaks in reports of stomach upsets in passengers departing from Spain. In contrast, reported stomach upset in passengers departing from Egypt peaked in the summer. Most of the cruises departing from Egypt were smaller river cruises, perhaps lessening the propensity for large norovirus outbreaks and presenting more frequent opportunities to go ashore. The high summer temperatures may have increased the likelihood of bacterial contamination of food or water either on board or ashore.

The decreasing trend observed in stomach upset reported on cruise ships is encouraging. Over the past ten years great effort has been made by cruise ship lines, Port Health Authorities and inspectorate organisations to improve the hygiene standards on ships. The cruise lines have sound business reasons for their continued interest in reducing the incidence of gastrointestinal illness on their ships. Furthermore, the US Vessel Sanitation Program inspection and scoring system has provided a strong incentive for ships travelling to the United States to maintain the highest hygiene standards. In September 2010, the European project for ship sanitation training network (SHIPSAN TRAINET) published the European Manual for Hygiene Standards and Communicable Disease Surveillance on Passenger Ships, and a ship inspection programme has been piloted [25]. It is hoped that with the strong collaboration of cruise lines, Port Health Authorities and national, trans-national and international organisations the incidence of infectious diseases, including gastroenteritis, will continue to fall from the current levels which are still higher than passengers would expect.

In this study it was not possible to produce a well fitted multivariable model to examine stomach upset. This is likely due to the limited information collected on the CSQs and the complex interplay of factors determining a person's risk of travel-related stomach upset. Behaviour choices, such as the types of meals consumed and how stringently hygiene advice is followed, have previously been shown to have an influence on the risk of travellers becoming ill while abroad on both land [3], [26] and cruise holidays [19], [27], and this level of detail was not available on the CSQs. Furthermore, for cruise ship travel, no details of the itinerary were available, so the start point of the cruise has been used for analysis of destinations. Finally, no information was available regarding whether cruise passengers stayed in land-based accommodation in the country of cruise departure prior to boarding the ship.

The systematic collection of CSQ data over many years has been instrumental in some major public health improvements in many holiday countries. It may be useful for the Travel Industry to discuss the methods for collection and use of this data with Public Health England to examine ways of improving the health benefits.
